# Entropy-Regulated Swelling as the Mechanistic Driver of Drug Diffusion in a Mechanically Robust Hydroxyapatite/PVA Hybrid Hydrogel

**DOI:** 10.1021/acs.macromol.5c03339

**Published:** 2026-02-05

**Authors:** Juliana Pretel de Souza, Vicente Lira Kupfer, Hugo Henrique Carline deLima, Jaqueline de Carvalho Rinaldi, Emerson Marcelo Girotto, Marcos Rogério Guilherme, Andrelson Wellington Rinaldi

**Affiliations:** † Rinaldi Research Group, Department of Chemistry, 42487 State University of Maringá – UEM, 5790 Colombo Avenue, 87020-900 Maringá, PR, Brazil; ‡ Postgraduation in Bioscience and Physiopathology – PBF, State University of Maringá – UEM, 5790 Colombo Avenue, 87020-900 Maringá, PR, Brazil

## Abstract

Hydrogels exhibit excellent permeability for solute transport, with their degree of swelling directly modulating drug diffusion through their polymer network. This dynamic hinders the quantitative prediction of the swelling mechanisms of these materials, owing to a decrease in configurational entropy resulting from the extension of polymer chains during water absorption. This study provides important insights into transport phenomena in a hydroxyapatite (HAp)–poly­(vinyl alcohol) (PVA) hydrogel by considering the thermodynamic principles governing molecular diffusion up to equilibrium and elucidating mechanisms relevant to drug delivery. HAp shows a hexagonal phase, and its unit cell volume increases by ∼2% after vinyl functionalization (HAp–π). PVA was converted to a chemically cross-linkable polymer and subsequently reacted with HAp–π to form a hybrid hydrogel network. The resulting system exhibits mechanical robustness resulting not only from chemical cross-links but also from noncovalent network constraints, which cooperatively give rise to a high density of effective cross-linking points. The hydrogel absorbs water and releases the drug slowly due to strong constraints imposed by the polymer structure. Despite these restrictions, molecular diffusion remains thermodynamically spontaneous (Δ*G* < 0), driven by a low, positive entropy change (Δ*S*), while enthalpic contributions (Δ*H*) are unfavorable. During swelling, water penetrates the hydrogel, driven by its higher chemical potential in the initially pure surrounding liquid, migrating into the polymer matrix and inducing network expansion, in a direction opposite to that of drug diffusion out of the hydrogel which further hinders the release dynamics because the solute is already in a high-entropy environment. Mass transport through a water-swellable release system constitutes an entropically driven process, dominated by diffusion within a constrained network. This work provides insight into entropy-regulated drug release, demonstrating that spontaneity is achieved at physiological temperature (∼37 °C) without altering the thermal energy so as to compromise long-term practical applications.

## Graphical abstract

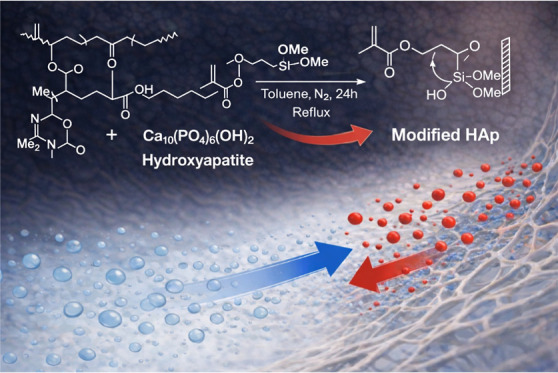

## 1 Introduction

Hydrogels have emerged as a prominent class of biomaterials owing to their remarkable swelling capacity, hydrophilicity, biocompatibility, and structural resemblance to biological tissues, which make them particularly attractive for drug delivery applications.^1–6^ These materials are synthesized from biological origin, semisynthetic, or synthetic polymers through physical or chemical cross-linking or polymerization reactions.^7–9^ Owing to their capacity to conform to diverse shapes, these systems can recover their functional properties,^10,11^ thereby restoring their original structure. However, the application of hydrogels in drug delivery systems remains constrained by two important factors.^1,12^ First, drug release is predominantly governed by water absorption, with the swelling capacity acting as the main determinant of diffusion into the surrounding liquid.^13,14^ Second, in their hydrated state, hydrogels typically exhibit low mechanical strength, limited elasticity, and poor energy dissipation, which can result in premature structural failure.^15–18^

Efficient theoretical diffusion-based models have been developed to predict the swelling mechanisms of these materials.^19,20^ However, quantitative predictions remain challenging due to the complex dynamics of water penetration into the hydrogel, which modulate the configurational entropy and hinder the development of advanced material-design and optimization strategies needed to enhance system performance. As swelling proceeds, polymer chains undergo progressive extension, reducing the number of accessible microstates and introducing an intrinsic entropic constraint to the system.^1,7,12,21,22^

The reliable behavior of water-swellable drug-delivery systems, in turn, requires a comprehensive understanding of the thermodynamic and kinetic processes that govern their behavior,^10,23^ along with an accurate evaluation of their mechanical performance.^16,24,25^

To investigate solute transport mechanisms relevant to drug delivery, poly­(vinyl alcohol) (PVA) was converted into a covalent hydrogel to provide a durable, mechanically stable network. These systems can readily support long-term applications suitable, unlike the physically assembled hydrogels, which are typically unstable and mechanically fragile, often undergoing a gel-to-solution transition. PVA is a synthetic polymer widely recognized for its suitability in biomedical applications due to its excellent biocompatibility,^25–27^ hydrophilicity,^28,29^ microporous network,^25,30^ favorable mechanical strength,^31,32^ and strong interfacial adhesion.^33,34^ To further tailor its physicochemical properties, trace amounts of *N*,*N*′-dimethylacrylamide (DMAA) were incorporated to increase the density of hydrophilic functional groups, thereby improving water affinity and facilitating gelation.^35,36^

In addition, hydroxyapatite (HAp), a calcium phosphate compound that mimics the mineral phase of bone,^37–39^ was introduced to reinforce the mechanical integrity of the hydrogel, with the intention of ensuring that the essential physicochemical and biological properties of the polymers are preserved. Cytotoxicity assays were performed as a preliminary assessment of biocompatibility to determine whether the inclusion of DMAA and HAp in the PVA matrix affected cellular viability or induced cytotoxic effects.

This study reports the preparation of a mechanically stable hydrogel based on a covalent HAp/PVA hybrid network that may, in principle, be reinforced by noncovalent constraints such as chain entanglements, polymer crystallite formation, hydrogen bonding, and inorganic phase–polymer interactions. Neomycin was used as a guest molecule that behaves as a mobile species within the polymeric network. To obtain a quantitative understanding of transport phenomena governed by water-absorption-driven release mechanisms, thermodynamic principles were applied to diffusion models as a guiding approach for designing drug delivery systems that maintain spontaneous release at physiological temperature (∼37 °C). If solute transport is favorable at a constant temperature, no additional heat is necessary, which ensures the system remains within temperature regimes relevant to its intended application. This modeling strategy does not attempt to reproduce classical thermodynamic derivations; rather, it draws on its fundamental concepts to elucidate the role of chemical potential gradients in the release system, capturing diffusion up to equilibrium and providing application-relevant insights.

## 2 Materials and Methods

The study utilized fetal bovine serum (Invitrogen); *N*,*N*-dimethylacrylamide (DMAA, 99%); penicillin/streptomycin/trypsin/EDTA (Gibco); poly­(vinyl alcohol) (PVA, 98–99% hydrolyzed, Mw 31–50 kg/mol, CAS 9002-89-5); glycidyl methacrylate (GMA, 97%); 3-(methacryloxypropyl)­trimethoxysilane (MPTS); neomycin trisulfate (all from Sigma-Aldrich); toluene (99.5%, Anhydrol); acetone (Synth); sodium persulfate (≥98%); sodium hydroxide (NaOH, 97%); hydrochloric acid (HCl, 37%); and calcium hydroxide (95%, Êxodo Científica). All reagents were of analytical grade and used as received.

### 2.1 Functionalization of Poly­(vinyl Alcohol) (PVA–π)

PVA (3.0 g) was dissolved in 100 mL of distilled water at 65 °C under stirring, and the pH was adjusted to 10.5 using a 1.0 mol L^–1^ sodium hydroxide solution. Subsequently, 2.4 mL of GMA was added to the stirred mixture. The system was sealed and allowed to react under continuous stirring for 24 h. After the reaction, the resulting material was precipitated in acetone to remove residual impurities, filtered under vacuum, cooled with liquid nitrogen, and freeze–dried for 24 h.

### 2.2 Synthesis of Hydroxyapatite (HAp)

A 0.3 mol L^–1^ phosphoric acid solution was gradually added to a 0.5 mol L^–1^ calcium hydroxide solution maintained at pH 8, which was adjusted using a 0.5 mol L^–1^ ammonium hydroxide solution. The resulting suspension was allowed to react at room temperature for 24 h. The solid product was then separated by centrifugation, thoroughly washed with deionized water, and dried at 80 °C for 12 h. Finally, the dried solid was calcined at 800 °C in a muffle furnace at a controlled heating rate of 3 °C min^–1^ for 8 h.

### 2.3 Functionalization of Hydroxyapatite (HAp–π)

HAp–π (9.0 g) was dispersed in 100 mL of toluene pretreated with silica (Zeolite A) at 25 °C under continuous stirring. Subsequently, 36 mL of MPTS was added to the mixture, and the reaction was carried out under reflux at 60 °C in a nitrogen atmosphere with vigorous stirring for 24 h. Then, the mixture was cooled to room temperature and centrifuged at 3500 rpm for 20 min. The resulting material was washed seven times with ethanol to remove residual impurities, incubated at 60 °C for 24 h to ensure complete drying, and finally subjected to reduced-pressure evaporation at 500 mmHg and 40 °C for 24 h to remove residual solvents.

### 2.4 Synthesis of the Pure and Hybrid Hydrogels

Known amounts of PVA–π or HAp–π were added to distilled water under continuous stirring. After homogenization, 0.2 μL of DMMA and 35 mg of sodium persulfate were introduced into the reaction medium. The mixture was subsequently transferred to a 10 mL cylindrical mold and heated at 65 °C until gelation was achieved. The compositions of the pure and hybrid hydrogels are presented in Table 1.

**Table 1:** –Summary of the Compositions of Pure and Hybrid Hydrogels^a^

samples	PVA-π g/mL H_2_O	HAp-π mg/mL H_2_O
H1	0.1	0.0
H2	0.2	0.0
H3	0.3	0.0
HH1	0.1	0.5
HH2	0.2	0.5
HH3	0.3	0.5

a The labels H and HH indicate pure and hybrid hydrogels, respectively.

### 2.5 Swelling Rate Measurements

The swelling behavior of the hydrogel was assessed by immersing 1 cm^3^ samples in 100 mL of deionized water at pH 5.5 and 7.4, maintained at 37 °C. At predetermined time intervals, the samples were removed, wiped off with absorbent paper to eliminate excess surface water, and weighed. This procedure was repeated until equilibrium swelling was reached. The swelling degree (*S*_w_) was calculated using eq 1, which relates the mass of the swollen hydrogel at time t (*M*_*t*_) to the mass of the dry hydrogel (*M*_*d*_)­

1 $$S_{w}=\frac{M_{t}-M_{d}}{M_{d}}$$

### 2.6 Mechanical Properties

Mechanical testing was conducted using a TAX.T2i texture analyzer fitted with a 5 kg load cell. Hydrogel samples were subjected to compressive deformation using a circular probe with a 0.5 mm diameter, applying a 1 mm displacement at a constant rate of 2 mm s^–1^. Prior to testing, the samples were equilibrated in water at 37 °C to ensure uniform hydration. Force–displacement data were recorded and used to construct stress (σ) vs strain (ε) curves.

### 2.7 Spectroscopic Characterization

Infrared vibrational spectra were obtained using a Thermo Fisher Scientific Nicolet IZ10 FTIR spectrometer. Measurements were conducted in the range of 4000–400 cm^–1^, with samples dispersed in spectroscopic–grade KBr. Each spectrum was obtained the average of 64 scans, recorded at a resolution of 4 cm^–1^, providing detailed information on functional group interactions and structural modifications within the hydrogel.

^1^H NMR spectra were obtained using a Bruker D8–Advance spectrometer operating at 300 MHz. D_2_O was used as the solvent, and measurements were performed at 60 °C using DMSO-*d*_6_ solutions of PVA and PVA–π. This analysis allowed the chemical modifications of the PVA backbone to be identified.

Structural analysis and phase determination of the hydroxyapatite and hybrid hydrogel components were conducted using wide-angle X-ray diffraction (WAXD). WAXD diffraction patterns of the inorganic materials were recorded on a Shimadzu LABX XRD-6000, using Ni-filtered CuK_α_ radiation at 40 kV and 30 mA. Diffraction data were collected with a 0.02° increment in 2θ at a scanning rate of 4° min^–1^.

### 2.8 Neomycin Release Experiments

Neomycin was loaded onto the hydrogel during the hydrogelation process at a concentration of 10% (w/w) relative to the total weight of the reactants. The neomycin-loaded hydrogels were immersed in 100 mL of an aqueous solution at pH 5.5 or 7.4, stirred at 50 rpm, and maintained at 37 °C. Drug release kinetics were analyzed using UV–vis spectrophotometry (Shimadzu UV mini 1240) at an absorbance wavelength of 360 nm. At predetermined intervals, 5 mL aliquots were withdrawn, and the neomycin concentration was determined using a calibration curve correlating absorbance with drug concentration.

### 2.9 Cytotoxicity Test

Cytotoxicity was assessed using VERO cell lines (African green monkey kidney epithelial cells) cultured at 37 °C in a 5% CO_2_ atmosphere. The cells were grown overnight in Dulbecco’s Modified Eagle Medium (DMEM; Gibco, MO, USA), supplemented with 10% (v/v) fetal bovine serum (FBS), and 1% penicillin/streptomycin. After reaching approximately 80% confluence, cells were detached using a 0.25% trypsin–EDTA solution, centrifuged, and resuspended in antibiotic-free DMEM. Cell density was adjusted to 2.0 *×* 10^5^ cells mL^–1^, corresponding to approximately 2.0 × 10^4^ cells per well, and 100 μL of the suspension was seeded into 96–well plates (TPP Switzerland). Cells were allowed to adhere for 24 h prior to treatment. Pure PVA and hybrid HAp/PVA hydrogels were UV-sterilized for 30 min on each side and dispersed in antibiotic-free DMEM at final concentrations of 1.0–1000 μg mL^–1^. Once adhered, the cells were washed twice with phosphate-buffered saline (PBS) and incubated with 100 μL of the hydrogel-containing media. Untreated cells cultured in DMEM served as the negative control (100% viability), while cells treated with 10% (v/v) dimethyl sulfoxide (DMSO) were used as the positive control for cytotoxicity. After 24 h of exposure at 37 °C in 5% CO_2_, cell viability was evaluated using MTT-based CellTiter 96 AQueous One Solution Cell Proliferation Assay (Promega, WI, USA). A volume of 20 μL of the reagent was added to each well, and the plates were incubated in the dark for 3 h. The formation of soluble formazan was quantified by measuring absorbance at 490 nm using an ASYS spectrophotometer (Biochrom, MA, USA). All experiments were performed in triplicate (*n* = 3) and repeated in three independent assays. Cell viability was expressed as a percentage relative to the negative control. Materials were considered noncytotoxic when cell viability exceeded 70%, in accordance with ISO 10993–5 standards.

## 3 Result and Discussion

### 3.1 Characterization of PVA Vinyl Functionalization by FTIR and ^1^H NMR

The modification of macromolecules with glycidyl methacrylate (GMA) may proceed via transesterification^40,41^ and/or epoxide ring-opening mechanisms,^41,42^ depending on the solvent system^43^ and reaction pH.^41^ In this study, PVA was modified under basic conditions (pH 10.5), where epoxide ring opening is the predominant pathway. Transesterification introduces methacrylate groups along the PVA backbone with glycidol as a removable byproduct, whereas epoxide ring opening yields covalently bound glyceryl methacrylate isomers through consumption of the epoxide ring (Figure S1).

The absorption bands at 1715 and 1714 cm^–1^ in the PVA–π spectrum (Figure 1a) correspond to the C=O stretching vibrations, and the prominent band at 1094 cm^–1^ is attributed to the intensified ester C–O stretching of the methacryloyl group linked to PVA hydroxyls. The band at 1177 cm^–1^, resulting from epoxide ring deformation, and the band at 1646 cm^–1^, characteristic of vinyl C=C stretching, are additional signals of the PVA–π formation.

**Figure 1:**
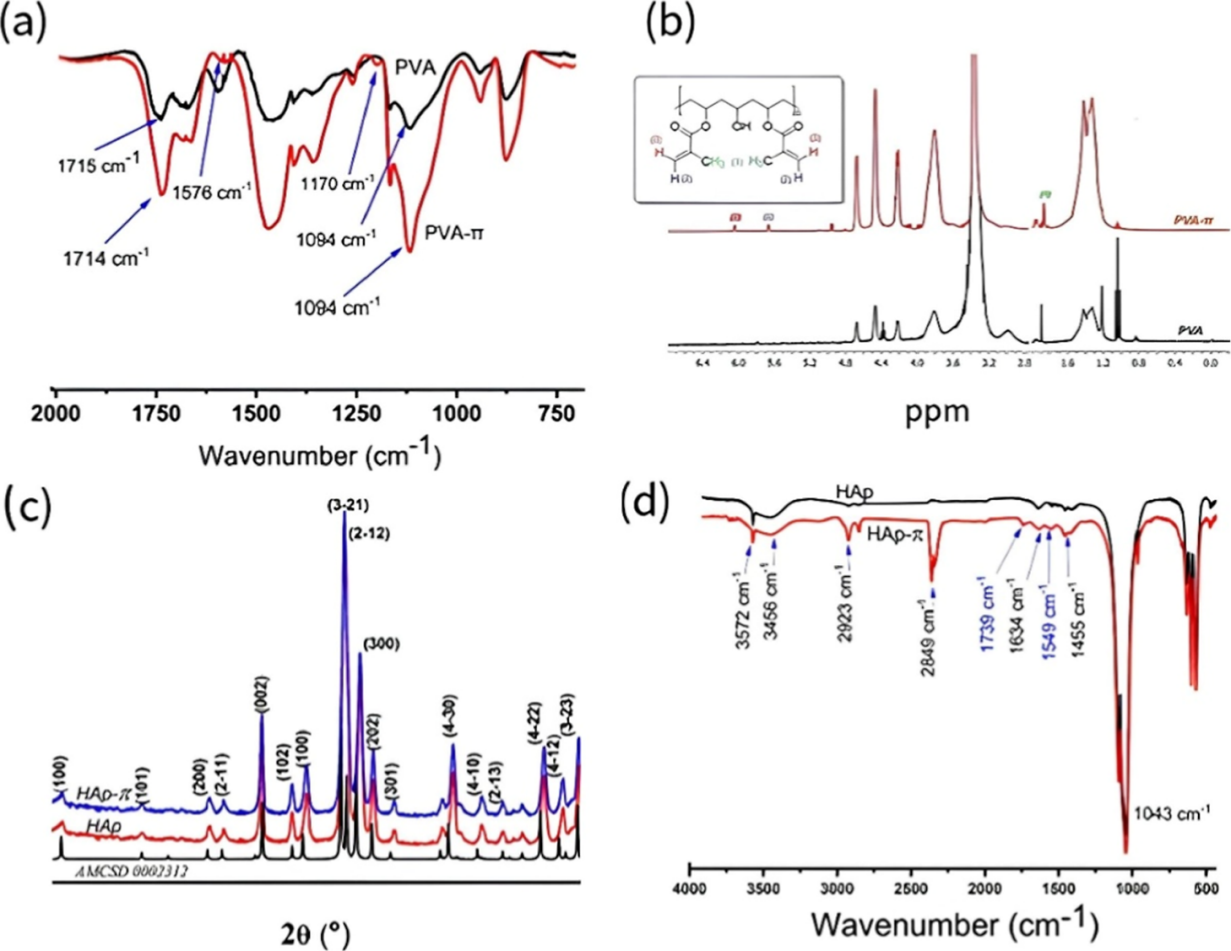
–FTIR and ^1^H NMR spectra of PVA and PVA–π (a,b) and XRD and FTIR patterns of HAp, and HAp–π compared with AMCSD 0002303 (c,d), showing surface vinyl functionalization and preserved crystallinity.

Figure 1b displays the ^1^H NMR spectra of PVA and PVA–π. The distinct signals at δ 6.05, δ 5.67, and δ 1.86 in PVA–π spectrum indicate the presence of methacryloyl functionalities derived from GMA. The peaks at δ 6.05 and δ 5.67 correspond to the vinyl protons (−CH=CH−), and the resonance at δ 1.86 is attributed to the methyl protons (−CH_3_) attached to the vinyl carbon. The resonances at δ 4.96 and 4.95 ppm are assigned to the vinyl protons of vinyl methacrylate units in PVA-π. The broad signal at δ ≈ 3.8 ppm arises from the methine proton (−CH­(OH)−) of the PVA repeat unit. The signals observed at 3.97–4.11 ppm are ascribed to groups of the glyceryl spacer^41^ formed upon epoxide ring opening of GMA and subsequent covalent attachment to the PVA backbone, as shown in Figures S2 and S3. The absence of characteristic epoxide-related resonances further supports complete epoxide ring consumption during the modification reaction. The absence of characteristic epoxide-related resonances further supports complete epoxide ring consumption during the modification reaction.

The degree of substitution (DS) was determined by ^1^H NMR spectroscopy (Figures S2 and S3) using the normalized integrals of the methacrylate vinyl protons (δ 5.67 and δ 6.05) as quantitative markers and the methine proton (−CH−) of the PVA repeat unit (δ ≈ 3.8) as the reference. DS was approximately 5%, corresponding to modification of approximately 5 PVA repeat units per 100. The number of chemically cross-linkable points (*x*_*c*_) was estimated from DS, assuming an average junction functionality of *f* = 4.^44^ The estimated *x*_*c*_ values for PVA-π, calculated using eqs S1–S5, can reach up to 10^21^, depending on the polymer content.

### 3.2 WAXD Analysis for the Structural Characterization of HAp

Figure 1c shows the WAXD patterns of HAp, HAp–π, and the crystallographic standard from the AMCSD (entry 0002303). The high-intensity diffraction peaks in the patterns, which are associated with the crystallographic planes (100), (101), (200), (2–11), (002), (102), (3–21), (2–12), (300), (202), (301), (4–30), (4–10), (2–13), (4–22), (4–12), and (3–23), correspond to higher-order reflections of the simple hexagonal *P*6_3_/*m* phase (SHP).

To gain further insight into the structure of prepared HAp, the WAXD data were fitted to a hexagonal-phase model (eq 2).^45,46^

2 $$\frac{1}{d_{hkl}^{2}}=\frac{4\left(h^{2}+hk+k^{2}\right)}{{3a}^{2}}+\frac{l^{2}}{c^{2}}$$

here, *a* and *c* are the lattice constants, and *d*_*hkl*_ is the interplanar spacing, which represents the positions of WAXD peaks according to Bragg’s law (eq 3).^47^

3 $$\lambda =2d_{hkl}\sin\theta$$

where λ is the wavelength of the incident radiation, and θ is the diffraction angle.

The parameters *a* and *c* were obtained by applying a least-squares fit to eqs 1 and 2. Using OriginPro (2019b version) software, the values obtained were a = 9,4 Å, and *c* = 6,8 Å. The unit cell volume of HAp was determined by considering that the SHP-like structure can be divided into equilateral triangles, according to following eq 4

4 $$V_{cell}=ach$$

here, *h* corresponds to the height of the triangle, which can be calculated using the Pythagorean theorem.

5 $$h^{2}=a^{2}-\frac{a^{2}}{4}=\frac{a\sqrt{3}}{2}$$

substituting *h* into eq 5 results in

6 $$V_{cell}=\left(\frac{a^{2}\sqrt{3}}{2}\right)c$$

The eq 6 defines the volume (*V*_cell_) of a primitive unit cell in a crystalline lattice. The calculated *V*_cell_ of the prepared HAp is 520.3 Å^3^, which is in agreement with the reference value of ∼528–530 Å^3^ reported in the literature.^48–71^ For HAp–π, *V*_cell_ increases slightly to 530.7 Å^3^, corresponding to an expansion of ∼2% relative to unmodified HAp. The presence of these defects can be further elucidated by integrating the primary WAXD peaks. The analysis reveals a slight reduction in peak intensities for HAp–π compared with unmodified HAp, with decreases of approximately 1–7% observed for the principal reflections ((100), (002), (211), (300), (202), and (310)). Although this trend is subtle and complicates a more detailed analysis of HAp–π formation by X-ray diffraction, FTIR analysis may provide more distinct insights into the modification process.

The FTIR spectra of HAp and HAp–π (Figure 1d) display the characteristic vibrational bands of hydroxyapatite, with the main phosphate (PO_4_^3–^) stretching vibration observed at 1043 cm^–1^ and the O–H stretching bands at 3572 cm^–1^ and 3456 cm^–1^, which are consistent with typical apatite structures. New absorption bands appear in the HAp–π spectrum at 1739 cm^–1^ and 1549 cm^–1^, attributed to the C=O stretching and C–O stretching vibrations, respectively, providing evidence of the reaction of the π-containing (MTPS) organic moieties with the HAp framework. The weak bands at 2923 cm^–1^ and 2849 cm^–1^, assigned to C–H stretching of aliphatic groups, give additional support of the presence of organic functionalities derived from the modifier. The reduction in band intensity in the 3000–3700 cm^–1^ range is attributed to the dehydration of Ca­(OH)_2_ during particle synthesis.

The reaction of MPTS with HAp proceeds via a nucleophilic substitution mechanism (Figure 2a). Surface hydroxyl groups (−OH) on HAp is supposed to attack the silicon atom (Si^4+^) of the MPTS methoxysilane moiety, shifting a methoxy group (−OCH_3_). This view is supported by the established hydrolysis–condensation mechanism of MPTS on HAp, in which surface hydroxyl groups react with hydrolyzed silanols to form Si–O–HAp linkages.^49,50^

**Figure 2:**
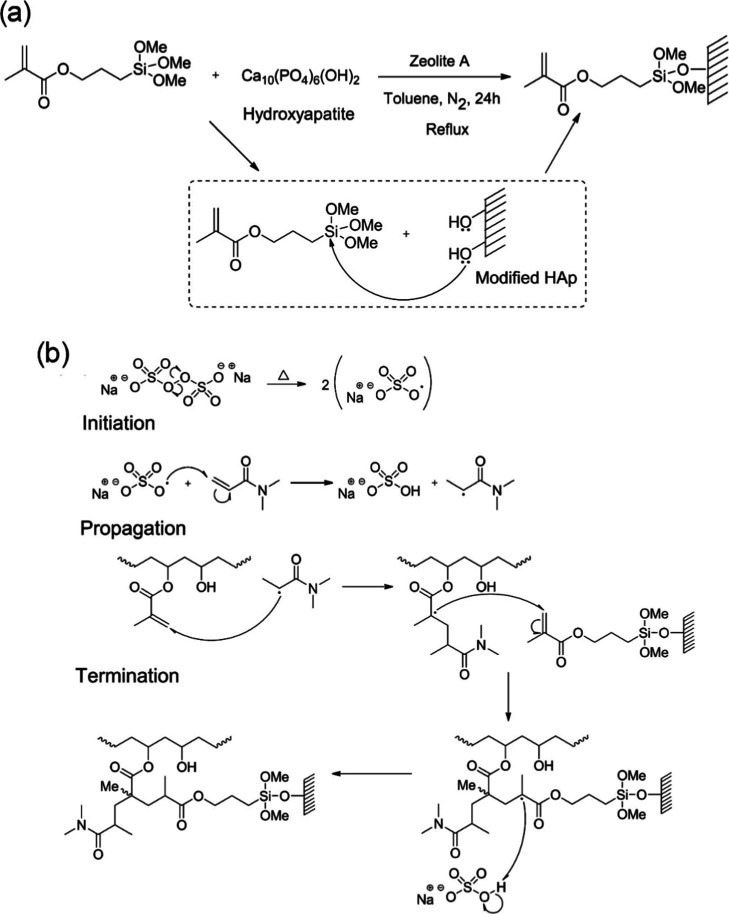
–Schematic representation of (a) the functionalization of HAp with MPS and (b) the radical reaction mechanism for hybrid hydrogel formation involving the vinyl groups of PVA–π, HAp–π, and DMAA.

The hydrogelation of the hybrid material occurs through a radical reaction involving the vinyl functionalities of PVA-π,^41^ HAp-π, and DMAA. The proposed mechanism for hybrid hydrogel formation (HH) is depicted in Figure 2b, including the stages of initiation, propagation, and termination.

### 3.3 Transport Water Mechanisms

At first approximation, the water absorption capacity of a polymer network system is governed by an osmotic process. The concentration gradient of polymer chains inside and outside the hydrogel, in combination with a flexible and hydrophilic polymer network, as well as the pH of the surrounding liquid, and presence of solutes, generates osmotic pressure,^1^ which serves as the driving force for water movement into the hydrogel. At equilibrium, the simultaneous inward and outward movement of water through the polymer network surface keeps the swollen hydrogel.

The Korsmeyer–Peppas semi–empirical model^51^ (eq 7) serves to describe the initial 60% of water absorption, during which swelling curves exhibit a linear progression over time. The diffusion exponent *n* characterizes the transport mechanism: When *n* = 0.45, the process is classified as Fickian transport, indicating that the water influx occurs at a slower rate than relaxation.

7 $$\frac{M_{t}}{M_{\infty }}=kt^{n}$$

For 0.45 < *n* < 1.0, the behavior represents an intermediate state between diffusion and relaxation. If *n* = 1.0, it indicates Case II transport, which occurs through a time-independent macromolecular relaxation mechanism. When *n* > 1.0, the water influx follows super Case II transport, resulting from the combined effects of diffusion, macromolecular relaxation, and polymer device erosion. For *n*0.45 (or <0.5,^52,53^ depending on geometry) the release curves show an initially linear regime limited to a small fraction of the total concentration change, after which the approach to equilibrium becomes markedly slower.

The *n* values in Table 2 are characteristic of *pseudo*-Fickian behavior, where solute release follows the relation *M*_*t*_/*M*_eq_ ∝ *t* ^*n*^ with *n* < 0.5. This transport regime reflects hindered water diffusion relative to classic Fickian behavior,^54^ arising from structural constraints such as high cross-linking density, increased diffusion pathway tortuosity, and restricted polymer chain mobility. Such behavior is associated with a lower-entropy state resulting from polymer chain extension during swelling.^1^ This phenomenon can be better understood in light of the statistical theory of polymer-chain elasticity. In this model, the configurational entropy is related to the mean end-to-end distance (*R*) of the chain, following the relation Δ*S* ∝ – *R* ^2^/*Nb* ^2^,^12^ where *b* denotes the effective bond length and *N* the number of statistically independent segments. During hydrogel swelling, polymer chains undergo progressive extension, which reduces the number of accessible microstates. An increase in the average *R* values of the PVA chains leads to a decrease in entropy.

**Table 2:** Diffusion Exponent (*n*) for Pure and Hybrid Hydrogels at Different pH Values

samples	pH 5.5	pH 7.4
H1	0.25(±0.01)	0.34(±0.06)
H2	0.28(±0.01)	0.38(±0.05)
H3	0.26(±0.03)	0.40(±0.02)
HH1	0.25(±0.01)	0.32(±0.02)
HH2	0.22(±0.04)	0.37(±0.05)
HH3	0.19(±0.02)	0.35(±0.01)

In addition, the *n* values are slightly higher at pH 7.4 than at pH 5.5, indicating a change in the water penetration profile within pure and hybrid hydrogels. HAp has the approximate chemical formula Ca_10_(PO_4_)_6_(OH)_2_, in which the phosphate anion (PO_4_^3–^) constitutes the central structural component. In aqueous media, phosphate originates from phosphoric acid (H_3_PO_4_), which does not exist as an isolated species, but rather participates in a series of acid–base equilibria: $H_{3}PO_{4}⇌H_{2}P{{O_{4}}_{4}}^{-}⇌{HP{{O_{4}}_{4}}^{2}}^{-}$ ⇌ PO_4_^3–^, with each chemical form predominating over a distinct pH range.^55^ The aqueous dissolution equilibrium of HAp involves the release of calcium, phosphate, and hydroxide ions according to Ca_10_(PO_4_)_6_(OH)_2(s)_⇌ 10Ca^2+^ + 6PO_4_^3–^ + 2OH^–^.^56^

Under acidic conditions, PO_4_^3–^ is progressively protonated to HPO_4_^2–^and $H_{2}{PO}_{4}^{-}$ , thereby shifting the equilibrium toward hydroxyapatite dissolution. By contrast, under mildly basic conditions PO_4_^3–^ is thermodynamically favored, promoting HAp stability, while the increased availability of OH^–^ supports occupation of hydroxyl sites within the HAp crystal lattice. Although PVA is not a strong polyelectrolyte, its affinity for water increases at pH 7.4, leading to enhanced chain hydration. Consequently, the greater swelling under mildly basic conditions arises predominantly from osmotic and electrostatic effects associated with the charged and stable HAp surface, together with increased hydration of the PVA matrix.

### 3.4 Mechanical Properties

The mechanical properties of the hydrogels were assessed by determining the elastic modulus (*E*), resilience (*U*_*r*_), and toughness (*T*). The results are presented in Table 3. The parameter *T*, which represents the energy absorbed per unit volume at rupture, reflects the extent of plastic deformation occurring at the point of fracture. *U*_*r*_ quantify the energy absorbed up to the yield point and characterize the extent of elastic deformation.

**Table 3:** –Mechanical Properties of Pure and Hybrid Hydrogels Determined at Equilibrium Swelling

samples	*E* (kPa)	*T* (kJ m^–3^)	*U*_r_ (kJ m^–3^)
H1	8.40(±0.24)	–	–
H2	23.81(±1.32)	14.56(±0.46)	7.45(±0.33)
H3	57.57(±2.52)	32.37(±1.54)	8.74 (±0.39)
HH1	29.15(±1.32)	23.96(±0.38)	8.32 (±0.14)
HH2	38.28(±1.69)	29.00(±0.43)	7.92 (±0.35)
HH3	70.33(±2.95)	34.84(±1.51)	10.64 (±0.47)

The H1 sample does not undergo mechanical rupture (*T*) during compression, which explains its low elastic modulus (*E*) of 8.4 kPa. Under the applied load, deformation occurs predominantly within the elastic regime, allowing full recovery of the original shape and dimensions upon unloading.^57^ An increase in PVA concentration leads to a pronounced stiffening response, characterized by an increase in the elastic modulus to 57.6 kPa for the H3 sample.

The combination of HAp-π with PVA-π improves mechanical performance, raising the modulus of each corresponding formulation (e.g., H2/HH2; H3/HH3). The hybrid hydrogels with higher PVA content show higher *T* values, reaching 29 kJ m^–3^ for HH2 and 34.84 kJ m^–3^ for HH3, indicating that greater mechanical energy is required for rupture. *U*_*r*_ is more prominent in HH3, indicating the higher energy recovery, whereas the other samples showed comparatively stable behavior.

Although no universal mechanical threshold defines robustness, performance criteria depend on the specific application,^58,59^ the number of elastically active chains (ν_*e*_) in the polymer network was estimated from the relationship between elastic modulus (*E*), shear modulus (*G*), and Poisson’s ratio (υ = 0.5 for hydrogels^60^), according to eqs S6–S10. The parameter *x*_*c*_, calculated from the conversion of double bonds in ^1^H NMR data, represents the number of covalent cross-linking points. The calculated values of ν_*e*_ and *x*_*c*_ are summarized in Table S1.

For clarity, both parameters were converted into dimensionless quantities to render them directly comparable and are therefore referred to as the “number of junction points”. This conversion was achieved by transforming the corresponding densities into absolute values using Avogadro’s number, as detailed in the Supporting Information.

The ν_*e*_ values are approximately 10^24^–10^25^, which is several orders of magnitude higher than the *x*_*c*_ values (∼10^20^–10^21^). The higher ν_*e*_ values arise from network connectivity and additional physical constraints that are not captured by *x*_*c*_. This mechanical reinforcement results not only from chemical cross-links but also from noncovalent network constraints, including chain entanglements, polymer crystallite formation, and hydrogen bonding.

### 3.5 Release Kinetics of Drug

Figure 3a,b shows the time-dependent drug release curves of pure and hybrid hydrogels at pH 5.5 and 7.4. The diffusion parameters for drug release are summarized in Tables 4 and 5. Release experiments were performed at 37 °C in PBS at pH 7.4, which simulates physiological plasma, and at pH 5.5 to assess the influence of pH on the diffusion mechanism.

**Figure 3:**
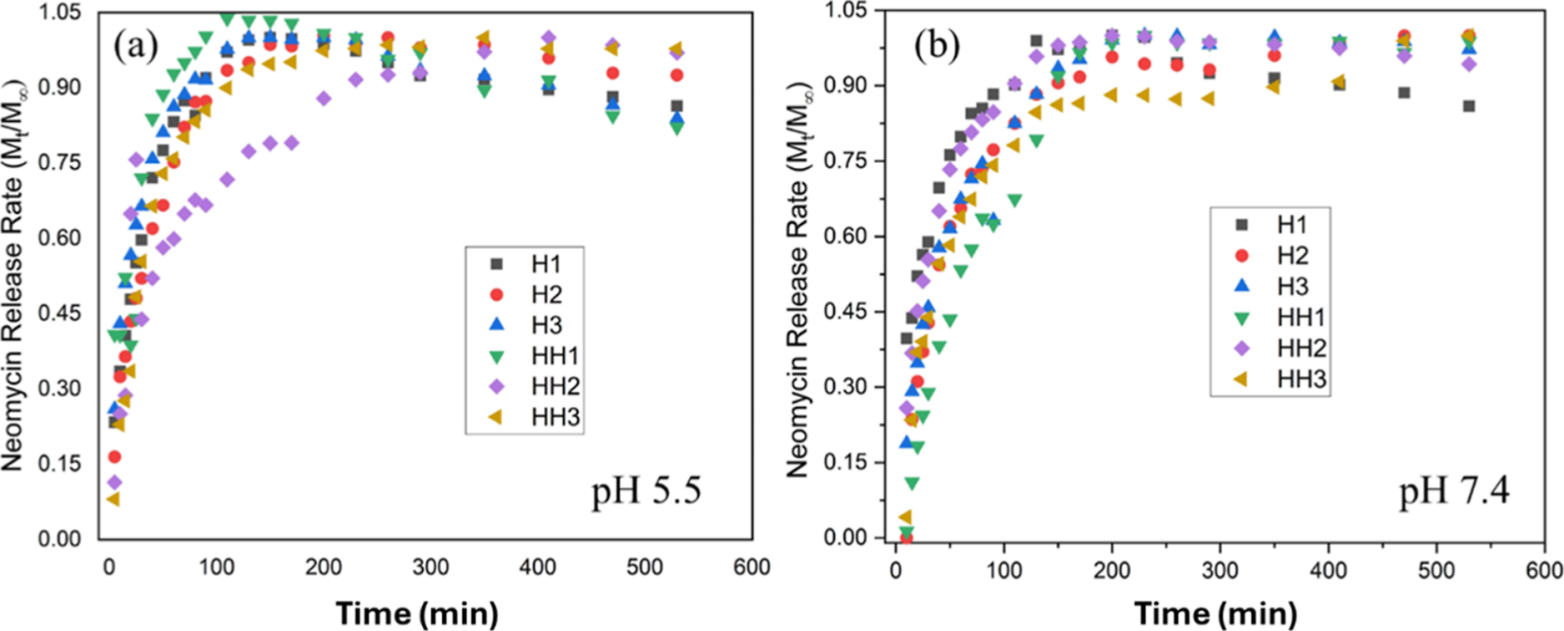
–Time-dependent neomycin release curves of pure and hybrid hydrogels at (a) pH 5.5 and (b) pH 7.4 until steady state is reached. The labels H and HH indicate pure and hybrid hydrogels, respectively.

**Table 4:** –Diffusion Parameters for Neomycin Release from Pure and Hybrid Hydrogels at pH 5.5

sample	*n*	*k*(μ s^–1^)	*R* ^2^	MDT(s)	*D*(*x10* ^6^ ) (cm^2^ s^–1^)
H1	0.49(±0.04)	436(±28)	0.991(±0.050)	1537(±61)	319(±13)
H2	0.51(±0.01)	360(±22)	0.997(±0.038)	1839(±49)	267(±10)
H3	0.36(±0.03)	609(±36)	0.990(±0.020)	1208(±72)	406(±22)
HH1	0.21(±0.02)	468(±22)	0.993(±0.058)	1765(±35)	278(±14)
HH2	0.51(±0.07)	395(±14)	0.981(±0.025)	1677(±40	293(±16)
HH3	0.99(±0.05)	460(±26)	0.972(±0.031)	1094(±83)	449(±25)

**Table 5:** –Diffusion Parameters for Neomycin Release from Pure and Hybrid Hydrogels at pH 7.4

sample	*n*	*k*(μ s^–1^)	*R* ^2^	MDT(s)	*D*(*x10* ^6^) (cm^2^ s^–1^)
H1	0.42(±0.03)	522(±31)	0.990(±0.029)	1350(±40)	363(±10)
H2	0.76(±0.08)	342(±19)	0.997(±0.025)	1661(±58)	295(±18)
H3	0.57(±0.03)	321(±24)	0.990(±0.045)	1987(±64)	247(±12)
HH1	0.94(±0.09)	248(±8)	0.990(±0.037)	2082(±76)	236(±9)
HH2	0.48(±0.04)	461(±3)	0.990(±0.033)	1464(±28)	335(±18)
HH3	0.37(±0.02)	379(±3)	0.989(±0.024)	1921(±55)	255(±21)

At pH 5.5, H3 exhibits diffusion-dominated (*pseudo*-Fickian) release behavior, as evidenced by the low ***n*** value of 0.36, whereas the relatively high ***k*** value reflects an increased initial diffusion rate without altering the underlying transport mechanism. This mechanism occurs when the system undergoes a slow progression toward equilibrium.

The H1 sample exhibits a higher ***k*** value, which is likely associated with a combination of factors, including its highly porous and low-density polymer network that facilitates diffusion. At pH 7.4, the hydrogels show a predominately drug release profile with a tendency to an anomalous mechanism. This mechanism arises when the hydrogel exhibits an intermediate behavior between pure diffusion and polymer relaxation, being strongly influenced by structural changes such as swelling degree, polymer chain relaxation, and matrix erosion.

Additional insights into the drug release mechanism can be obtained by calculating the mean dissolution time (MDT) and the drug diffusivity coefficient (*D*) from the values of *n* and *k*. MDT defines the average duration for a drug molecule to be released from a gel and can be obtained by applying eq 8 ^61^

$$MDT=\left(\frac{n}{n+1}\right)k^{\left(-1/n\right)}$$ 8

The parameter *D* represents the effective diffusivity of the drug within the matrix. It provides information on the intrinsic ability of the drug to move through the hydrogel. High *D* values correspond to faster molecular movement, resulting in low MDT and more rapid release. Accordingly, *D* and *MDT* are inversely proportional and can be related through the eq 9

$$D\approx \frac{a^{2}}{MDT}$$ 9

here, *a* represents to the characteristic dimension of the hydrogel (cm).

A hydrogel is viewed as a macromolecular system in which solid and liquid phases coexist in a steady state,^3^ forming microscopic pathways through which the drug must diffuse to be released. *D* reflects how drug molecules move through these pathways, and MDT indicates the average time required for release. When the pathways are well hydrated and less obstructed, diffusion is faster (high *D*) and release occurs more rapidly (low MDT). Conversely, more restricted pathways slow diffusion (low *D*) and prolong release (high MDT).

The magnitude of the diffusion parameters MDT and *D* indicates extremely slow drug release, as shown in Tables 4 and 5. Comparing H3 and HH3 at pH 5.5 (Table 4), the *n* value increases from 0.36 to 0.99, indicating a significant change in the release mechanism. Although drug diffusion within these PVA-rich hydrogels is more restricted due to structural constraints, this condition promotes an enhanced concentration gradient and faster release into the surrounding pH 5.5 medium, as evidenced by the high *D* and low **MDT** values.

For H3, an increase in pH enhances chain hydration (Table 5), thereby facilitating drug diffusionppa through the hydrogel. This effect shifts transport from *pseudo*-Fickian toward anomalous behavior, as indicated by the increase in ***n***, the decrease in ***D***, and the corresponding increase in **MDT**.

In contrast, HH3 exhibits an inversion of the release mechanism with increasing pH (Table 5): under acidic conditions, a relaxation-controlled profile (*n* ≈ 1) dominates due to a strong concentration gradient. At high pH, polymer–HAp hydrogen bonding restricts chain mobility, increases tortuosity, and reduces effective diffusivity, leading to *pseudo*-Fickian transport (*n* ≈ 0.37). The high ***D*** value observed at pH 5.5 compared with that at pH 7.4 for HH3 indicates that solute diffusion within the hydrogel becomes less efficient in a basic medium, effectively inverting the dominant release mechanism.

### 3.6 Thermodynamics of Drug Release

The thermodynamic parameters were obtained on the basis of the experimental drug-release data. In aqueous macromolecular systems, the chemical potential (μ) serves as the primary quantity and starting point for addressing physicochemical mass-transport phenomena, including mixing, dissolution, equilibrium, and diffusion.^62^ In this context, the release process at the hydrogel interface is governed by differences in **μ**.^63,64^

The following sequence of equations aims to link the chemical potential within the hydrogel (**μ** ^**sol**^), which is directly influenced by the presence and spatial distribution of the drug within the polymer matrix, to the chemical potential of the initially pure external solvent (**μ** ^**Liq**^).

The purpose of this sequence is not to reproduce classical thermodynamic derivations, but to clarify the physical basis for applying chemical potential gradients to the specific release system studied here, describing drug diffusion at equilibrium (Figure S4).

The expression for **μ** ^**sol**^ is given by

10 $$\mu^{sol}=\mu^{Liq}+RT\ln a_{A}$$

where a _***A***_ is the absolute activity, *R* is the universal gas constant (8.314 J·K^–1^·mol^–1^), and *T* denotes the absolute temperature of the system. In nonelectrolyte solutions, the thermodynamic behavior is described in terms of the difference between the chemical potential in the solution phase (**μ** ^**sol**^) and the reference liquid state (**μ** ^**Liq**^). By rewriting eq 10 in the following form

11 $$\mu^{sol}-\mu^{Liq}=RTln\frac{a_{A}}{a_{A}^{0}}$$

The value of the standard activity a _***A***_^0^ is defined as 1 mol·L^–1^ for solutions. Since the absolute activity has units of mol·L^–1^, the ratio o a _***A***_/ a _***A***_^0^ is dimensionless and is referred to simply as the activity (***a***_***i***_). Thus, eq 11 can be rewritten as

12 $$\mu^{sol}-\mu^{Liq}=RTln{\;a}_{i}$$

The activity a _***i***_ in eq 12 represents the effective concentration of a species, accounting for intermolecular interactions that contribute to the establishment of thermodynamic equilibrium. When a _***i***_ → 1, the condition **μ** ^**sol**^ = **μ** ^**Liq**^ is satisfied, and the release rate becomes negligible. As the release progresses, the amount of drug in the solution and the corresponding thermodynamic properties vary (**μ** ^**sol**^ **< μ** ^**Liq**^), requiring an adaptation of the classical equilibrium equations to account for nonideal behavior.

From the fundamental thermodynamic relation for a single-component system (eq 13).^63,64^

dG=−SdT+VdP 13

where ***S*** and ***V*** are the system entropy and volume, respectively. Since ***G*** = ***G*** (***T***, ***P***), the total differential of *G*, considering the contributions of temperature and pressure, can be expressed by eq 14

14 $$dG={\left(\frac{\partial G}{dT}\right)}_{p}dT+{\left(\frac{\partial G}{dP}\right)}_{T}dP$$

For a system with more than one component, ***G*** = ***G*** (***T***, ***P***, ***n***_1_, ***n***_2_...), an additional term is introduced into the differential equation, which then becomes

$$dG={\left(\frac{\partial G}{dT}\right)}_{P,n_{i}}dT+{\left(\frac{\partial G}{dP}\right)}_{T,n_{i}}dP+\overset{n}{\underset{i=1}{\sum }}{\left(\frac{\partial G}{{\partial n}_{i}}\right)}_{T,P}{dn}_{i}$$ 15

where ***dn***_***i***_ corresponds to the infinitesimal variation in the molar amount of that component. Controlled release experiments are typically conducted under isothermal (37 °C) and isobaric conditions; therefore, eq 15 simplifies to the following form

$$dG=\overset{n}{\underset{i}{\sum }}{\left(\frac{\partial G}{{\partial n}_{i}}\right)}_{T,P,}{dn}_{i}$$ 16

Considering that the derivative ${\left(\partial G/{\partial n}_{i}\right)}_{T,P}=\mu_{i}$ , eq 16 yields

17 $$dG=\sum_{i}^{n}\mu_{i}{dn}_{i}$$

Integrating eq 17 with respect to ***dn***_***i***_ at constant temperature and pressure gives

18 $$G-G^{*}=\sum_{i}^{n}\mu_{i}\left(n_{i}-n_{i}^{*}\right)$$

By defining ***n***_i_^*^ = 0 and ***G*** * = 0, the initial boundary of the system is established, where the composition begins with reference to a baseline free energy level. Consequently, eq 18 reduces to the form

19 $$G=\sum_{i}^{n}\mu_{i}n_{i}$$

For a mixing process, the free energy is given by Δ ***G***_**mix**_ = ***G***_**final**_ – ***G***_**initial**_ and the chemical potential can be expressed as **μ**_***i***_ − **μ**_***i***_^0^. Substituting these terms into eq 19 yields

20 $${\Delta G}_{mix}=\sum_{i}^{n}n_{i}\left(\mu_{i}-\mu_{i}^{0}\right)$$

By establishing the equivalence between **μ** ^**sol**^ – **μ** ^**Liq**^ in eq 12 and (**μ**_***i***_ – **μ**_***i***_^0^) in eq 20, Δ ***G***_**mix**_ can be written as a logarithmic function of a _***i***_, yielding eq 21 ^64^

21 $${\Delta G}_{mix}=nRT\sum_{i}^{n}a_{i}\ln a_{i}$$

For the controlled release system, where mixing results from variations in the amount of drug within the aqueous phase, the activity can be defined as a _**H**20_ + a _**drug**_ = 1, or equivalently a _**H**20_ = 1− a _**drug**_. Given that equilibrium thermodynamic and kinetic properties are not physically equivalent, particular care was taken in the present analysis to restrict consideration to cumulative release data obtained only after the equilibrium is reached. The equilibrium release regime is characterized by a deviation from linearity in the diffusion curve after a given time, at which point the drug release rate (υ_**rel**_) equals the solute absorption rate (υ_**abs**_), establishing a dynamic diffusion equilibrium (***υ***_**rel**_ ⇌ ***υ***_**abs**_),^20^ as indicated in Figure S4. Under these conditions, the extracted parameters describe the rates of inward and outward fluid and solute transport within the hydrogel system. As a result, diffusion becomes stabilized and a thermodynamic equilibrium state is established, in which the rates of drug release and absorption in both the hydrogel and surrounding solvent phases remain constant.

Accordingly, by considering the ratio ***M***_***t***_/***M***_**∞**_ as the drug activity at equilibrium, ((***M***_***t***_/***M***_**∞**_)/***dt*** = 0), the mixing free energy Δ ***G***_**mix**_ for a nonideal solution (eq 21)^63^ becomes the Gibbs free energy of release (Δ ***G***_**rel**_).

$${\Delta G}_{rel}=n_{t}RT\left[\left(\frac{M_{t}}{M_{\infty }}\right)\ln\left(\frac{M_{t}}{M_{\infty }}\right)+\left(1-\frac{M_{t}}{M_{\infty }}\right)\ln\left(1-\frac{M_{t}}{M_{\infty }}\right)\right]$$ 22

where ***n***_***t***_ is the total number of moles. The entropy of release (Δ ***S***_**rel**_) is obtained by differentiating eq 22, resulting in eq 23

23 $${\Delta S}_{rel}=-n_{t}R\left[\left(\frac{M_{t}}{M_{\infty }}\right)\ln\left(\frac{M_{t}}{M_{\infty }}\right)+\left(1-\frac{M_{t}}{M_{\infty }}\right)\ln\left(1-\frac{M_{t}}{M_{\infty }}\right)\right]$$

Knowing the values of Δ ***G***_**rel**_ and Δ ***S***_**rel**_, the enthalpy of release­(Δ ***H***_**rel**_)­can be determined using the Gibbs–Helmholtz relation (eq 24).^63,64^

24 $${\Delta G}_{rel}={\Delta H}_{rel}-T{\Delta S}_{rel}$$

A system is at equilibrium when its free energy reaches a minimum under specified conditions of temperature, pressure, volume, and composition. On a macroscopic scale, this state is characterized by the persistence of all system properties over time, with no observable changes occurring indefinitely.

Table 6 shows the thermodynamic parameters for neomycin sulfate release at pH 5.5 and 7.4. The negative Δ ***G***_**rel**_ values indicate that the release process is favorable at the pH values studied. For comparison, when Δ ***G***_**rel**_ is positive, additional energy is required to induce a spontaneous state transition, which may limit the applicability of the drug delivery system under physiological conditions.

**Table 6:** –Thermodynamic Parameters for Neomycin Sulfate Release from Pure and Hybrid Hydrogels

	pH 5.5			pH 7.4		
Samples	Δ*G* (kJ)	Δ*S* (JK^–1^)	Δ*H* (kJ)	Δ*G* (kJ)	Δ*S* (JK^–1^)	Δ*H* (kJ)
H1	–1.099(±0.032)	31.7(±1.4)	∼8.7^–^	–0.778(±0.042)	17.4(±0.8)	∼4.6
H2	–0.753(±0.021)	29.5(±1.0)	∼8.4	–0.667(±0.037)	21.7(±0.6)	∼6.1
H3	–1.208(±0.038)	34.8(±1.7)	∼9.6	–0.256(±0.025)	8.5(±1.1)	∼2.4
HH1	–1.301(±0.070)	37.5(±1.5)	∼10.3	–0.215(±0.021)	8.10.81(±0.7)	∼2.3
HH2	–0.309(±0.021)	8.8(±1.4)	∼2.4	–0.197(±0.014)	9.6(±1.5)	∼2.8
HH3	–0.302(±0.011)	9.3(±0.8)	∼2.6	–0.147(±0.012)	4.3(±0.5)	∼1.2

Although the experiments were conducted under isothermal conditions (37 °C), as required for physiological systems, the calculated enthalpy parameters provide valuable insight into the release mechanism. The unfavorable Δ ***H***_**rel**_ values observed in the hydrogels indicate that macromolecule-solvent and macromolecule-HAp interactions, as well as intermolecular and intramolecular attractive forces, are weak. In addition, drug–matrix interactions are negligible, as the solute behaves as a guest molecule. Its transport occurs primarily by diffusion through the hydrogel network, with mobility progressively regulated by polymer-chain relaxation and network hydration during water penetration, as evidenced by the effective diffusivity (***D***) of the drug within the matrix.

The spontaneity of the release process arises from an entropic contribution. The unfavorable Δ ***H***_**rel**_ and low, positive Δ ***S***_**rel**_ values indicate that diffusion proceeds slowly and is primarily governed by the structural constraints of the hydrogel network. Notably, the slightly positive Δ ***S***_**rel**_ values indicate spontaneous rearrangements of the macromolecular chains, allowing the network to retain a certain degree of conformational mobility. During swelling, water molecules penetrate the hydrogel, driven by their higher chemical potential in the initially pure surrounding liquid,^65^ migrating toward the polymer network. Although water molecules diffuse rapidly, their penetration into the hydrogel is regulated by the cooperative expansion of the polymer network, which occurs at a significantly slower rate.^66^ Consequently, simultaneous mass transport into and out of the hydrogel is dominated by spontaneous diffusion through a constrained network.

From the perspective of molecular phenomena, and by analogy with regular solutions,^63^ the surrounding liquid possesses entropy corresponding to the number of microstates accessible to its molecules.^62,63^ The presence of drug dissolved within the hydrogel introduces an additional entropic effect, acting as a retarding factor for drug release, since the solute is already in an environment of high entropy.

The hybrid hydrogels with high PVA contents (HH2 and HH3) exhibit slightly reduced Δ ***H*** values, resulting from polymer–polymer, polymer–water, and polymer–HAp interactions, such as hydrogen bonding and coordination-type interactions. In these hydrogels, drug release remains entropy-driven.

### 3.7 Cytotoxicity Tests

Cell viability (%CV) was determined according to eq 25, and the corresponding results are presented in Table 7.

25 $$\%CV=\left(\frac{A_{t}}{A_{c}}\right)\times 100$$

where *A*_*t*_ is the absorbance of cells treated with test sample and *A*_*c*_ is the absorbance of the control (untreated cells).^67^ The results indicate that both the pure PVA hydrogel (H3) and the hybrid HAp/PVA hydrogel (HH3) are highly cytocompatible over the full tested concentration range (1–1000 μg·mL^–1^), underscoring their potential for safe biomedical applications. After 24 h of exposure, the cytotoxicity assay showed that VERO cells maintained over 99% viability after 24 h of exposure to high concentrations of the samples. At the low concentration, cell viability exceeded 100%, indicating no in vitro toxicity. At lower concentrations (≤500 μg mL^–1^), cell viability values slightly exceeded 100%. Such behavior is often attributed to normal biological variability and mild metabolic stimulation rather than genuine proliferation.^68^ To more accurately assess cell viability, the *p*-value was calculated to determine the probability that observed differences between treated and control cells occurred by chance.^69^ Since *p* > 0.05, the treatment did not significantly affect cell viability.

**Table 7:** Evaluation of Cytotoxicity for Pure PVA (H3) and Hybrid HAp/PVA (HH3) Hydrogels in VERO Cells After 24 h Exposure, Shown as Percentage of Cell Viability Relative to Untreated Control

% cell viability						
samples	1000 μg/mL	500 μg/mL	250 μg/mL	125 μgmL	1 μg/mL	IC_50_
H3	99.3	104.0	101.2	103.1	102.2	>1000 μg/mL
HH3	100.7	102.6	100.8	103.2	103.1	>1000 μg/mL

## 4 Conclusions

This work provides mechanistic insights into transport phenomena within HAp–PVA hydrogels by combining thermodynamic analysis with water absorption–driven models relevant to drug-delivery performance under equilibrium conditions. Structural characterization confirmed the formation of a hexagonal HAp phase. PVA-π was reacted with HAp–π to produce a covalent hydrogel. DS of PVA-π was approximately 5%, as determined by ^1^H NMR spectroscopy. Based on DS, the number of chemically cross-linkable points was estimated to be on the order of ∼10^20^ to 10^21^, and the number of effective cross-linking points, estimated from the elastic modulus, was substantially higher, on the order of 10^24^ to 10^25^. This enhanced mechanical robustness arises not only from chemical cross-links but also from noncovalent network constraints, including chain entanglements, polymer crystallite formation, and hydrogen bonding, which collectively promote a high density of effective cross-linking points. The hydrogels absorb water and release drug slowly, regulated by spontaneous diffusion (Δ*G*_rel_ < 0) through a constrained network. Considering the unfavorable Δ*H*_rel_ and low, positive Δ*S*_rel_ values, the transport through the hydrogels is governed by an entropically driven mechanism. In addition, the increase in PVA content in the hybrid hydrogels leads to slightly reduced Δ*H* values, resulting from polymer–polymer, polymer–water, and polymer–HAp interactions. The pure PVA hydrogel and the hybrid HAp/PVA hydrogel are highly cytocompatible, underscoring their promise for safe biomedical applications.

## Supplementary Material



## Data Availability

Data will be made available on request.

## Notes

All data necessary to support the conclusions of this work are provided, including derivations of *x*_*c*_ and ν_*e*_, supplementary schematics and tables, ^1^H NMR data, diffusion equilibrium plots, and additional references (PDF)
