# Supplementary material for: Entropy-Regulated Swelling as the Mechanistic Driver of Drug Diffusion in a Mechanically Robust Hydroxyapatite/PVA Hybrid Hydrogel
Source: Macromolecules. 2026 Feb 5;59(4):2230–41. doi: 10.1021/acs.macromol.5c03339 (PMC12947675; doi:10.1021/acs.macromol.5c03339)
Supplement: Supplementary file 1 [file ma5c03339_si_001.pdf]

Entropy–Regulated Swelling as the Mechanistic Driver of Drug Diffusion in a Mechanically Robust Hydroxyapatite/PVA Hybrid Hydrogel

Juliana Pretel de Souza,[a] Vicente Lira Kupfer,[a] Hugo Henrique Carline de Lima,[a] Jaqueline de Carvalho Rinaldi,[b] Emerson Marcelo Girotto,[a] Marcos Rogério Guilherme,[a*] Andrelson Wellington Rinaldi[a*]

[a]Rinaldi Research Group, Department of Chemistry, State University of Maringá – UEM, 5790 Colombo Avenue, 87020–900, Maringá–PR, Brazil.

[b]Postgraduation in Bioscience and Physiopathology – PBF, State University of Maringá – UEM, 5790 Colombo Avenue, 87020–900, Maringá–PR, Brazil.

**Corresponding author:* awrinaldi@uem.br mrguilherme@uem.br

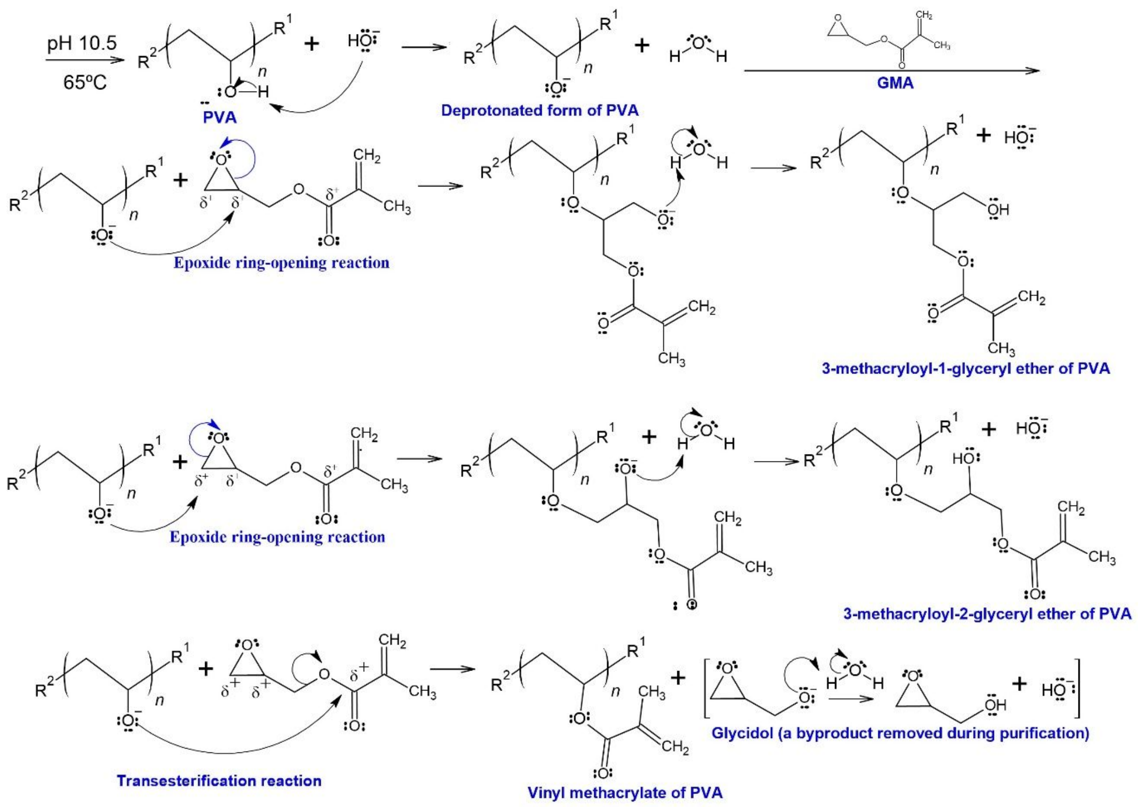

Figure 1S - Schematic representation of the reaction between GMA and PVA at pH 10.5, yielding vinyl methacrylate–substituted PVA via transesterification and 3-methacryloyl-1- and 3-methacryloyl-2-glyceryl ether–PVA via epoxide ring opening.

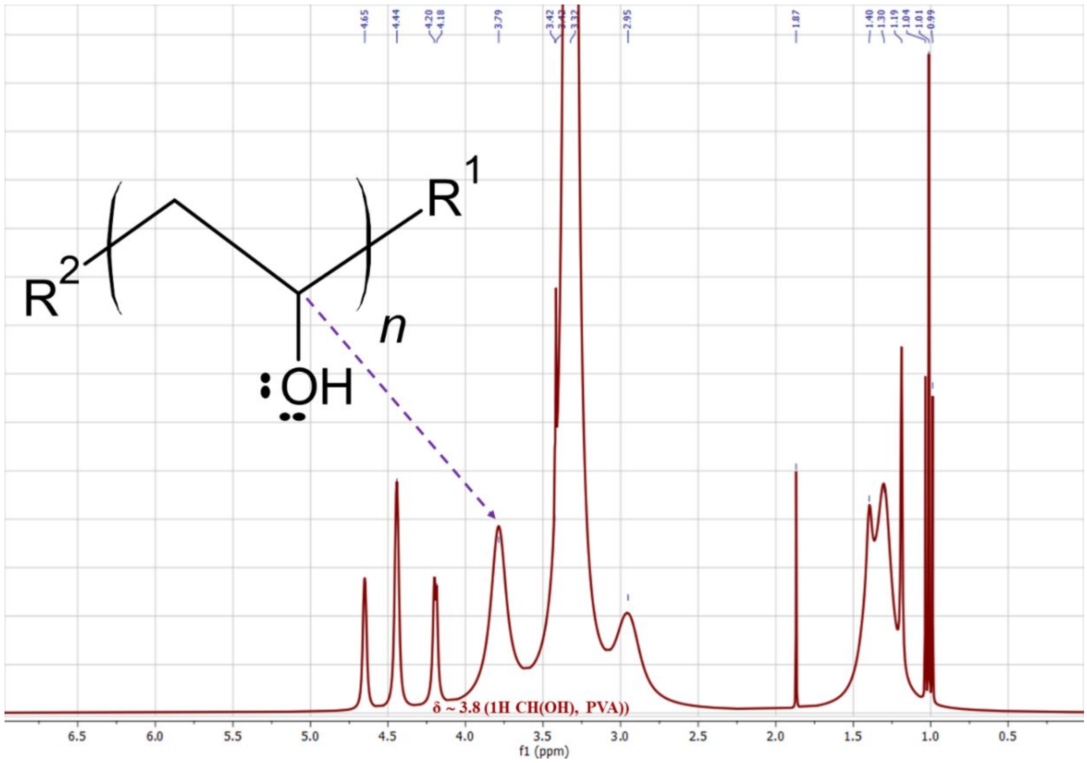

Figure 2S - ¹H NMR spectrum of unmodified PVA The broad signal at δ ≈ 3.8 ppm arises from the methine proton (–CH(OH)–) of the PVA repeat unit.

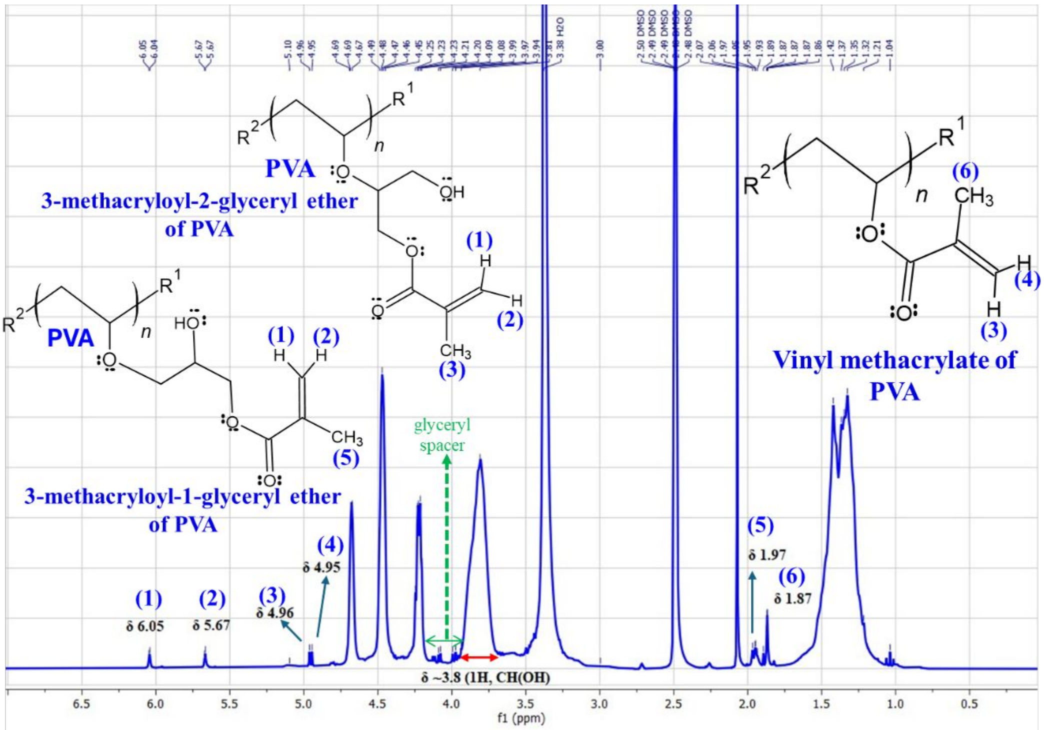

Figure 3S - ¹H NMR spectrum of modified PVA showing the formation of 3-methacryloyl-1-glyceryl ether of PVA, 3-methacryloyl-2-glyceryl ether of PVA, and vinyl methacrylate of PVA (300 MHz, DMSO-d_6). The signals at δ 6.05 and 5.67 ppm correspond to vinyl protons of the methacryloyl groups in the 3-methacryloyl-1- glyceryl ether and 3-methacryloyl-2-glyceryl ether moieties, while the signal at δ 1.97 ppm is assigned to the methyl protons of the methacrylate group.[1] The resonances at δ 4.96 and 4.95 ppm are attributed to the vinyl protons of vinyl methacrylate units grafted onto PVA. The broad signal at δ ≈ 3.8 ppm arises from the methine proton (–CH(OH)–) of the PVA repeat unit.[2]

The number of chemically crosslinkable points (x_c) was estimated from DS, assuming an average junction functionality of $f = 4$.[3]

Considering that each vinyl group on a chain connects two crosslinking points and therefore has two chain ends, the following relationship applies.

$$2N = fx_c \quad \text{(S1)}$$

where N is the number of elastically effective chains and x_c is the number of crosslinking junctions. By setting $f = 4$ and rearranging, the expression becomes:

$$x_c = \frac{2N}{f} = \frac{N}{2} \quad \text{(S2)}$$

Relating N to the mass of the dry polymer (m_{PVA}) used in the hydrogel synthesis and to the molar mass of the PVA repeating unit ($MM_{PVA} = 44{,}05\ g\ mol^{-1}$), yields:

$$N = \frac{m_{PVA}}{MM_{PVA}} \quad \text{(S3)}$$

By substituting Equation (S3) into Equation (S2), the following expression is obtained:

$$x_c = \frac{m_{PVA}}{2MM_{PVA}} \quad \text{(S4)}$$

The values of x_c in PVA is obtained introducing the number of Avogadro ($N_A = 6.023x10^{23}mol^{-1}$) and the degree of substitution ($DS = 0.05$) into Equation (S4).

$$x_c = \frac{m_{PVA}}{2MM_{PVA}}(N_A * DS) \quad \text{(S5)}$$

For comparison, this parameter was also independently obtained from elastic modulus (E) measurements. The relationship between elastic modulus (E), shear modulus (G), and Poisson's ratio (μ) is well established for isotropic materials[4] through Equation (S6):[4,5]

$$E = 2G(1 + \mu) \quad \text{(S6)}$$

For hydrogel $\mu = 0.5$,[6] Equation (S6) is rewritten as

$$E = 3G \quad \text{(S7)}$$

The parameter G can be obtained using Treloar's approach,[7] which estimates the shear modulus from the number density of elastically active chains in the polymer network.[3]

$$G = \nu_e RT \quad \text{(S8)}$$

where ν_e is the number density of elastically active chains in the polymer network, R is the ideal gas constant, and T is the absolute temperature of the system. Given that the elastic modulus is known in the present work, equations (S7) and (S8) can be combined to yield the following expression:

$$G = \frac{E}{3} = \nu_e RT \quad \text{(S9)}$$

To obtain the number of effective crosslinking points, the equation is rewritten in terms of ν_e and multiplied by N_A:

$$\nu_e = \frac{E}{3RT} N_A \quad \text{(S10)}$$

The x_c values were determined by fitting the experimental ^{1}H NMR data to Equation 5S. In parallel, the ν_e values were obtained from fits of the elastic modulus data to Equation 10S. The resulting parameters are summarized in Table S1.

Table S1– Effective number of chemically crosslinkable points (x_c), and effective number of crosslinking points (ν_e).

Samples	x_c	ν_e
H1	$3.42x10^{20}$	$2.04x10^{24}$
H2	$6.82x10^{20}$	$5.78x10^{24}$
H3	$1.02x10^{21}$	$1.39x10^{25}$
HH1	$3.42x10^{20}$ (*)	$7.08x10^{24}$
HH2	$6.82x10^{20}$(*)	$9.30x10^{24}$
HH3	$1.02x10^{21}$(*)	$1.71x10^{25}$

(*) Because the same amount of PVA was used to prepare both pure and hybrid hydrogels, the corresponding x_c values remain unchanged for each sample pair (H1/HH1, H2/HH2, and H3/HH3).

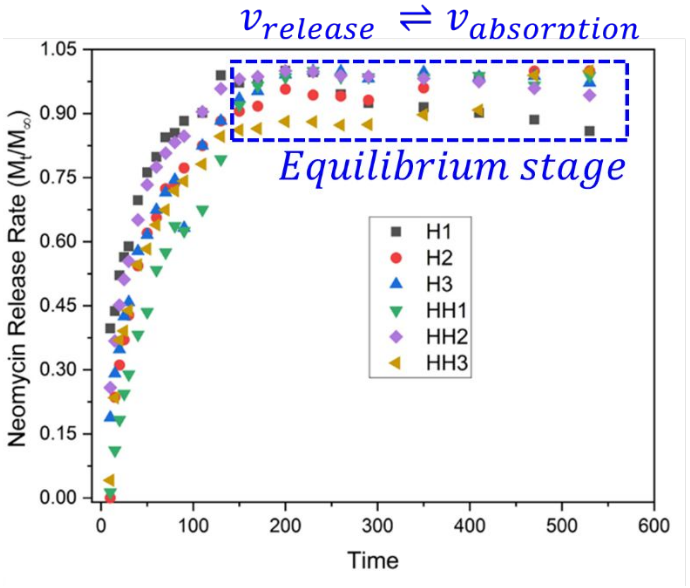

Figure S4 – Time-dependent neomycin release profiles for PVA and hybrid hydrogels, highlighting the equilibrium release stage. At equilibrium ($v_{rel} \rightleftharpoons v_{abs}$), the rates of drug release and absorption in both the hydrogel and solvent phases remain constant.

(1) van Dijk-Wolthuis, W. N. E.; Kettenes-van den Bosch, J. J.; van der Kerk-van Hoof, A.; Hennink, W. E. Reaction of Dextran with Glycidyl Methacrylate: An Unexpected Transesterification. *Macromolecules* 1997, *30* (11), 3411–3413. https://doi.org/10.1021/ma961764v.

(2) Reis, A. V.; Fajardo, A. R.; Schuquel, I. T. A.; Guilherme, M. R.; Vidotti, G. J.; Rubira, A. F.; Muniz, E. C. Reaction of Glycidyl Methacrylate at the Hydroxyl and Carboxylic Groups of Poly(Vinyl Alcohol) and Poly(Acrylic Acid): Is This Reaction Mechanism Still Unclear? *Journal of Organic Chemistry* 2009, *74* (10), 3750–3757. https://doi.org/10.1021/jo900033c.

(3) Richbourg, N. R.; Peppas, N. A. The Swollen Polymer Network Hypothesis: Quantitative Models of Hydrogel Swelling, Stiffness, and Solute Transport. *Prog. Polym. Sci.* 2020, *105*, 101243. https://doi.org/10.1016/j.progpolymsci.2020.101243.

(4) Callister, W. D. . *Materials Science and Engineering: An Introduction*; John Wiley & Sons, 2006.

(5) Tschoegl, N. W.; Knauss, W. G.; Emri, I. Poisson's Ratio in Linear Viscoelasticity – A Critical Review. *Mech. Time. Depend. Mater.* 2002, *6* (1), 3–51. https://doi.org/10.1023/A:1014411503170.

(6) Yang, P.; Boer, G.; Snow, F.; Williamson, A.; Cheeseman, S.; Samarasinghe, R. M.; Rifai, A.; Priyam, A.; Elnathan, R.; Guijt, R.; Quigley, A.; Kaspa, R.; Nisbet, D. R.; Williams, R. J. Test and Tune: Evaluating, Adjusting and Optimising the Stiffness of Hydrogels to Influence Cell Fate. *Chemical Engineering Journal* 2025, *505*, 159295. https://doi.org/10.1016/j.cej.2025.159295.

(7) Treloar, L. R. G. *The Physics of Rubber Elasticity*, Third Edition.; Treloar, L. R. G., Ed.; Oxford University Press: New York, 1975.
